# Assessment Tools for Executive Function and Adaptive Function Following Brain Pathology Among Children in Developing Country Contexts: a Scoping Review of Current Tools

**DOI:** 10.1007/s11065-021-09529-w

**Published:** 2021-12-06

**Authors:** Kwabena Kusi-Mensah, Nana Dansoah Nuamah, Stephen Wemakor, Joel Agorinya, Ramata Seidu, Charles Martyn-Dickens, Andrew Bateman

**Affiliations:** 1 Department of Psychiatry, University of Cambridge, Clifford Allbutt Building Cambridge Biomedical Campus CB2 OAH, Cambridge, UK; 2 Komfo Anokye Teaching Hospital, P. O. Box 1934, Kumasi, Ghana; 3 Pantang Hospital, Accra, Ghana; 4 Accra Psychiatric Hospital, Accra, Ghana; 5 School of Health and Social Care, University of Essex, Colchester, UK; 6 Department of Psychiatry, University of Michigan Health System, 1500 E Medical Center Dr, Ann Arbor, 48109 MI, USA

**Keywords:** Executive function, Adaptive function, Assessment, Psychometrics, Developing countries, Children

## Abstract

Several tools have been developed to assess executive function (EFs) and adaptive functioning, although in mainly Western populations. Information on tools for low-and-middle-income country children is scanty. A scoping review of such instruments was therefore undertaken.

We followed the Preferred Reporting Items for Systematic Review and Meta-Analysis- Scoping Review extension (PRISMA-ScR) checklist (Tricco et al., in *Annals of Internal Medicine 169*(7), 467–473, 2018). A search was made for primary research papers of all study designs that focused on development or adaptation of EF or adaptive function tools in low-and-middle-income countries, published between 1^st^ January 1894 to 15^th^ September 2020. 14 bibliographic databases were searched, including several non-English databases and the data were independently charted by at least 2 reviewers.

The search strategy identified 5675 eligible abstracts, which was pruned down to 570 full text articles. These full-text articles were then manually screened for eligibility with 51 being eligible. 41 unique tools coming in 49 versions were reviewed. Of these, the Behaviour Rating Inventory of Executive Functioning (BRIEF- multiple versions), Wisconsin Card Sorting Test (WCST), Go/No-go and the Rey-Osterrieth complex figure (ROCF) had the most validations undertaken for EF tests. For adaptive functions, the tools with the most validation studies were the Vineland Adaptive Behaviour Scales (VABS- multiple versions) and the Child Function Impairment Rating Scale (CFIRS- first edition).

There is a fair assortment of tests available that have either been developed or adapted for use among children in developing countries but with limited range of validation studies. However, their psychometric adequacy for this population was beyond the scope of this paper.

**Supplementary Information:**

The online version contains supplementary material available at 10.1007/s11065-021-09529-w.

## Introduction

### Background

Executive function is a modern complex concept that has only recently begun to find a common consensus of what exactly it means. Since Lezak first coined the term “executive functions” (EF) (Lezak, 1983) and subsequently defined it as “those capacities that enable a person to engage successfully in independent, purposive, self-serving behaviour” (Lezak et al., 2004), the concept has undergone several evolutions to its current understanding. In the early 2010’s, Diamond defined executive function as “top-down control processes” of human behaviour (Diamond, 2013) whose primary function was “supervisory control” as posited by earlier researchers (Stuss & Alexander, 2000). Diamond went on to describe three core or basic executive functions: **Inhibition**, **Working Memory** and **Cognitive Flexibility** (also called ‘set/task shifting’). They (Diamond, 2013) and others then went on to posit that these three core functions are combined in different ways to achieve what they described as the "higher executive functions": **Planning** (referring to the ability to identify and organize the steps and elements needed to achieve a goal; Diamond, 2013; Lezak et al., 2004), **Problem-solving**, and **Logical and abstract reasoning.** To back up their model, Diamond refers to work done by Miyake and colleagues (Miyake et al., 2000) and by Lehto et al. (Lehto et al., 2003) who both independently showed by factor analysis that three latent factors emerged (Diamond, 2013). This 3-core-factor model has been fairly well replicated (Collins & Koechlin, 2012; Hall & Marteau, 2014; Karbach & Unger, 2014; Lunt et al., 2012; Miyake & Friedman, 2012).

Adaptive function is a broad concept that covers an array of physical and social functioning- including communication, motor skills and daily living skills. But in the context of cognitive functioning, adaptive function may be viewed as the practical expression of executive functions in an everyday functional context. It may be defined as the ability to carry out everyday tasks within age and context appropriate constraints (World Health Organization, 2001), which may also be impaired following brain injury (Simblett et al., 2012). In the broader context of physical functioning, many chronic conditions can result in impairment of adaptive function. However, in this present study, the term shall be restricted to the narrow scope of adaptive function following brain injury or brain pathology.

#### Measuring Executive and Adaptive Functions in The Context of Developing Countries: The Effect of Culture and Socioeconomic Status

Until recently, many researchers seemed to take for granted that the concept of “executive functions” *would* be understood in largely the same way by all people-groups. The seminal work of Lev Vygotsky in the 1930’s (Vygotsky, 1986 English translation) makes a profound point about the dangers of simply “translating” a term meant to convey a specific concept developed in a particular culture into another language and culture without first checking for conceptual equivalence in that recipient language’s culture when working cross-culturally. To that point, several effects of socio-cultural differences on EF have been noted in the literature such as age-matched children and adolescents in Hong Kong out-performing their UK counterparts on all EF functions when controlling for all other relevant factors (Ellefson et al., 2017), with similar differences among immigrants in Denmark (Al-Jawahiri & Nielsen, 2020), and among an indigenous Mayan community in Mexico compared to urban controls (Ostrosky-Solís et al., 2004) plausibly attributable to cultural differences.

Similarly, regarding socioeconomic status (SES) and EFs, a recently published study among pre-schoolers from South Africa and Australia reported surprisingly that a highly disadvantaged South African subsample from Soweto out‐performed middle‐ and high‐SES Australian pre‐schoolers on two of three EFs (Howard et al., 2020), suggesting the possibility of EF‐protective practices within low-and-middle-income countries. In contrast, in a study from the United States, chronic exposure to poverty was predictive of young children’s poor performance on measures of executive function (Raver et al., 2013) suggesting that the impact of SES on EFs is complex and may depend on the stage of development among other factors. Even more so than with EF, social contexts and cultural expectations affect adaptive function because they shape whatever children learn and perform (Law, 2002; Poulsen & Ziviani, 2006).

But in all these speculations of plausible explanations for these observed differences, one cannot draw the conclusions typically drawn on the effect of culture, SES and other factors on the true underlying latent variable of EF or adaptive function if the thorny issue of using tools developed in foreign cultures to obtain cross-cultural measurement is not adequately addressed (Gannotti & Handwerker, 2002). Thus, assessment of item bias and measurement invariance of any tool used in a cross-cultural context is a crucial but oft-ignored step in the comparison of any two groups, for meaningful conclusions to be made (Fischer & Karl, 2019).

Therefore, a review of assessment tools that, rather than simply assuming universal applicability of any tool developed anywhere, have specifically been either developed or purposefully adapted to a developing country context using scientifically robust methods, is a worthwhile endeavour for practitioners and researchers in such contexts.

#### Rationale for Present Study

Improved healthcare has led to reduced mortality rates among children under five years in developing countries (Bakare et al., 2014). However, whether these children who survive are thriving adequately is in some doubt. Increasingly, the disease burden among children in low-and-middle-income countries is shifting from the so-called "childhood killer diseases" to other chronic conditions which may lead to significant impairment and morbidity but not outright mortality (Abubakar et al., 2016; Kieling et al., 2011). Several of these conditions may lead to neurobehavioral difficulties which affects brain function as well as the mental health and well-being of these children. These are often described under the catch-all term of "Acquired Brain Injury" (Bennett et al., 2005; Stuss, 1983). These conditions can lead to frontal lobe dysfunction which encompasses EF and adaptive function but is often neglected (Simblett et al., 2012).

Better EF is linked to many positive outcomes (Diamond, 2013) such as greater success in school (Duncan et al., 2007; St Clair-Thompson & Gathercole, 2006), while deficits in EF are associated with slow school progress (Morgan et al., 2017) difficulties in peer relationships (Tseng & Gau, 2013) and poor employment prospects (Bailey, 2007). Behaviourally, EF deficits may manifest as distractibility, fidgetiness, poor concentration, chaotic organization of materials, and trouble completing work (Bathelt et al., 2018). Given the difficulties seen, it is therefore important that mental health and rehabilitation services are able to pinpoint areas of greatest difficulty and target interventions appropriately and cost effectively through accurate assessments (Simblett et al., 2012).

Several tools have been developed to assess these areas of frontal lobe functioning in various populations. However, most of these assessment tools have been developed for mainly Western or high-income country populations with not much being known about the tools available for assessing children from low-and-middle-income countries. A literature search revealed only one recently published scoping review on the subject (Nyongesa et al., 2019), which while being very commendable only focused on tools for adolescents (excluding school-age children), searched a very limited scope of databases (only 3) and did not particularly focus on tools developed or adapted specifically for low-and-middle-income countries. Given the high burden of infections and neurodevelopmental conditions in low-and-middle-income countries (Bitta et al., 2017; Merikangas et al., 2009) which are known causes of acquired brain injury which affects EF and adaptive function, awareness of appropriate assessment tools for EF and adaptive function in this specific context and among a wide age-range of children will be highly desirable for clinicians and researchers in these settings.

A Scoping Review approach was chosen in this study because unlike a typical systematic review (which aims to answer a specific question about a specific population according to a rigid set of a priori delimiting factors detailed in a protocol), a scoping review has a broader interest, with the general aim of mapping out the literature and addressing a broader research question, but with the same level of rigour as a systematic review (Shamseer et al., 2015). A scoping review is also unlike a traditional systematic review in that a critical appraisal or risk of bias assessment would be done in a systematic review, while not done in a scoping review (Tricco et al., 2018). Therefore, because the question of interest here was not about the rigour of evidence of one specific tool, or class of tools, used in one specific population within a specific context, but rather just a broad overview of all potential tools used in a broadly defined context, a scoping review was better suited than a systematic review for the research questions specified below.

#### Objectives

The present study seeks to undertake a scoping review of published literature to systematically map out whether there are purpose-built assessment tools for executive and adaptive functioning among children (including adolescents) in low-and-middle-income country contexts, and if not, which developed-country tools have been adapted or validated for use among the population of interest, as well as document any knowledge gaps that may exist. The following research questions were therefore formulated:

-
  1. What tools for executive function and adaptive functioning following brain pathology have been adapted or developed or validated for use among children in low-and-middle-income country contexts?
  2. Which of these tools have the most literature published supporting their validation for use among children and adolescents in low-and-middle-income countries?

In this paper, we do not aim to critically appraise and summarise the evidence for the scientific rigour of the methodologies used, or report on the psychometric measurement properties established for the identified instruments, as this is beyond the scope of a scoping review. A systematic review of EF and adaptive function measurement tools in low-and-middle-income countries will however be reported on in a subsequent paper.

### Methods

This scoping review was conducted following the Preferred Reporting Items for Systematic Review and meta-analysis- Scoping Review extension (PRISMA-ScR) reporting checklist (Tricco et al., 2018), with our protocol also being drafted using the PRISMA- Protocol extension (PRISMA-P) 2015 guideline (Shamseer et al., 2015). While a specific protocol for this scoping review could not be pre-registered on the dedicated systematic reviews protocols repository PROSPERO (it was rejected on grounds that PROSPERO only registers systematic reviews and not scoping reviews), the protocols for the subsequent systematic reviews of EF and adaptive function measurement tools that proceeded from this scoping review *have* been successfully registered on the PROSPERO website (see here: https://www.crd.york.ac.uk/prospero/) with registration numbers CRD42020202190 and CRD42020203968 for the EF tools systematic review and adaptive function tools systematic review respectively. Since the papers that were critically appraised in these subsequent systematic reviews were initially identified following essentially the same protocol used for this scoping review, the reader’s attention is drawn to the pre-registration details of these systematic reviews as an accurate documentation of the pre-registered methodology that was followed for this scoping review as well.

#### Eligibility Criteria

We considered papers of all study designs (qualitative, quantitative, and mixed methods papers) that focused mainly on the target outcomes (executive functioning or adaptive functioning following brain pathology) among children in low-and-middle-income countries. The paper also had to primarily be concerned with developing, adapting or assessing the validity of the instrument of choice as one of its main stated study aims (if not the main), and not just as an incidental concern, to be eligible. Peer-reviewed articles, as well as expert opinions and published guidelines with provided rationales were considered. Participants considered were children aged 5 years and above to 18 years, including both healthy and clinical populations.

All studies recorded in the databases searched that were published at any time were considered, with no year limitations being placed on the search. In operational terms, this meant that papers published from 1^st^ January 1894 (the earliest date in PsychINFO, one of the databases searched) to 15^th^ September 2020 (the last day of update of the search strategy) were included. With respect to settings, studies conducted in developing country settings were selected. “Developing country” was defined using the World Bank list of lower-income countries (LIC), and middle-income countries (including both lower-middle-income countries-LMIC and upper-middle-income countries-UMIC) defined as of 2012 (Cochrane Library, 2012; World Bank Group, 2019) which are collectively referred to as “low-and-middle-income countries”. In terms of language, no a priori language limitations were placed on the search. Full articles written in English, and those in other languages that could reasonably be translated (either by using Google Translate or by finding native speakers willing to volunteer their services) were included. An appendix of potentially relevant articles in other languages that could not be translated is provided in Appendix II.

Specifically excluded, apart from those which did not generally meet the inclusion criteria above, were: (a) studies that only used the instrument as an outcome measurement instrument (for instance in randomized controlled trials) rather than specifically evaluating its psychometric properties or reporting on its local adaptation, (b) studies in which the EF or adaptive function instrument was used in a validation study of another (non-EF or adaptive function) instrument (i.e., validation was **not** of the EF or adaptive function tool, but rather another instrument for another construct such as say “long term memory”), (c) news articles, blog posts and other such mass media outlet writings, (d) studies on animals, and (e) studies focused on high income countries.

#### Information Sources

The following databases were searched for the following reasons:

1. MEDLINE (OVID interface, 1946 onwards), because this is one of the largest databases for health and medical literature (also known as “pubmed”) maintained by the United States government.
2. EMBASE (OVID interface, 1974 onwards), because it is complementary to MEDLINE and also because it is mainly a pharmacology and pharmaceuticals database it might have had validation papers related to drugs targeting frontal lobe dysfunction.
3. Cochrane library (current issues), since it is the main database of systematic reviews. Also, this was included because both Cochrane library and EMBASE include not only just published journal articles but also unpublished data (“grey literature”) like conference proceedings, and drug repository databases etc.
4. PsychINFO (1894 onwards), because it is the main database for psychiatry and psychology related research.
5. Global health (1973 onwards), this is a database for public health focused articles which might include eligible validation papers in the context of epidemiological surveys.
6. Scopus, since this is a database dedicated to multidisciplinary research (including qualitative research), given that we were interested in capturing research of a multidisciplinary nature.
7. Web of Science, since this is a database dedicated to multidisciplinary research
8. SciELO, this is Latin America focused database providing scholarly literature in sciences, social sciences, and arts and humanities published in leading open access journals from Latin America, Portugal, Spain, and South Africa; this was an important source of non-English language studies from low-and-middle-income countries in Latin America, particularly from Brazil.
9. Education Resources Information Centre (ERIC, 1966 onwards), important for papers on cognitive assessment published in the special education literature; included theses, dissertations, and teaching guides.
10. British Education Index (BEI, 1996 onwards), because it covers research done in education on evaluation and assessment, technology, and special educational needs.
11. Child Development & adolescent studies (CDAS, 1927 onwards), for same reason as above. Included theses, dissertations, and teaching guides.
12. Applied Social Sciences Index and Abstracts (ASSIA), because it is an important source for multidisciplinary papers. Includes social work, nursing, mental health and education journals.

### Gray Literature Data Sources

13. Open grey (1992 onwards). Includes theses, dissertations, and teaching guides
14. PROSPERO. Repository of pre-registered study protocols for systematic reviews for trial protocols for similar scoping reviews through PROSPERO.
15. Cochrane library (see above)
16. EMBASE (see above)
17. ERIC (see above)
18. CDAS (see above)

Thus 14 unique databases were searched. The initial search was done by 20^th^ March 2020 while the final updated search was completed by 15^th^ September 2020. Further, to make sure we were thorough in our literature search, we also scanned the reference list of selected papers for other papers of possible interest which might have been missed in the literature search, particularly so for systematic and scoping review papers we found in our search. The search in the ‘grey literature’ was included to enable us capture unpublished and non-peer-reviewed data on the matter. This was to help mitigate the risk of publication bias and other meta-biases.

#### Search Strategy

We developed literature search strategies using text words and medical subject headings (MeSH terms- including using the ‘explode’ function to get all related sub-categories of the MeSH terms) related to the following themes:

- Executive function/Frontal lobe function/Frontal lobe damage/Adaptive Function and their variants using truncation
- Assessments/Validation/reliability/norms/reproducibility/standardization of instruments and their variants using truncation
- Children/adolescents and their variants using truncation
- Developing countries/lower-middle-income-countries and their variants using truncation

The search strategy was developed by a member of the study team (KKM) who had undergone extensive training in conducting Systematic Reviews and in using search strategies in all the above-named databases from the Medical Library Services. The search strategy was also reviewed by an experienced Medical Librarian who has extensive expertise in systematic review searching. The full search strategy for MEDLINE is re-produced in Appendix I (see Supplemental Material).

#### Selection of Sources of Evidence

**Fig. 1:**
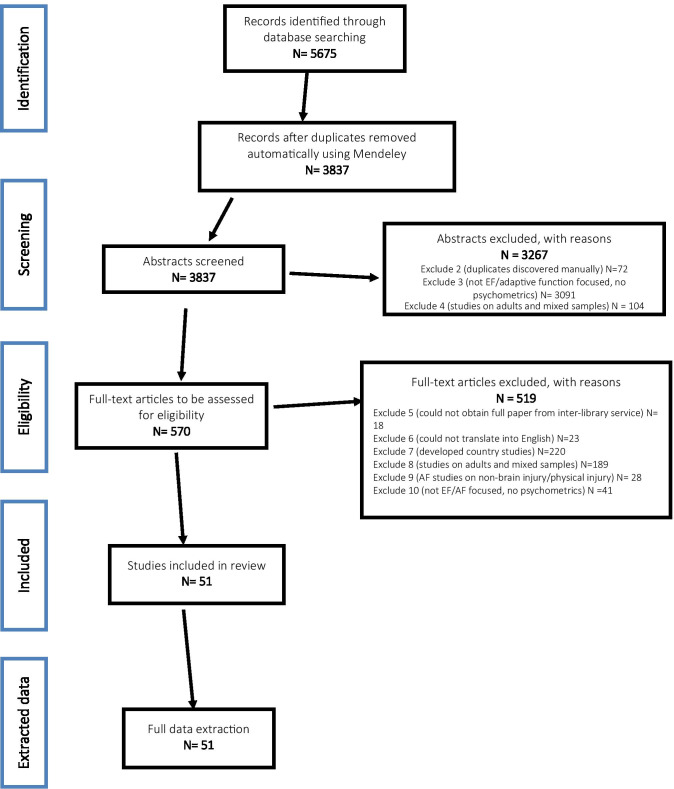
PRISMA flow diagram of study selection

For the selection process six reviewers worked on all abstracts and full papers. At the screening phase, at least two independent reviewers screened each title and abstract obtained from the search and compared their results with each other. Where there were disagreements on eligibility based on abstract alone, the full text article was retrieved and reviewed, and all discrepancies discussed and resolved. Where resolution was not possible after discussion, a third independent reviewer was brought in as arbiter, and as a last resort, the guarantor was consulted as a final arbiter. Further, the pre-resolution inter-rater agreement was calculated and reported as ranging between 81.6%—88.9%, which was above the recommended minimum 80% agreement. In accordance with PRISMA recommendations, the selection process was documented in a flow diagram (see Fig. 1 below).

#### Data Charting Process

At least two reviewers independently extracted data from the screened articles using a purpose-made data extraction chart, designed using items from the PRISMA-ScR and PRISMA-P checklists, to select suitable articles meeting the inclusion criteria. Table 1 in Appendix II shows a sample of this chart. First a calibration exercise was done for all 6 reviewers using a sample of 100 abstracts and 10 full paper articles to ensure uniform use of the screening criteria and the charting forms. The search results were uploaded and saved into Mendeley using the ‘*groups’* function, which allowed online collaboration and discussion among the reviewers. Study abstracts and full texts were uploaded based on the screening criteria. Duplication were minimized by employing the ‘*merge duplicates*’ function on Mendeley. In-spite of this, manual de-duplication still had to be done for a few abstracts. The Data were charted using Excel spreadsheets.

**Table 1:** Database Results and Dates

**Database**	**Date of search coverage**	**Initial search** by 20/03/20	**Initial de- duplication**	**Updated search** by 15/09/20	**Updated de- duplication**
MEDLINE via OVID	1946—05/09/20	803	662	939	760
EMBASE^a^	1974—05/09/20	27	26	29	27
Cochrane Library^a^	^*^11/09/20	67	49	68	48
PsychINFO	1894—11/09/20	1314	985	1490	812
Global Health	1973—11/09/20	38	14	38	13
Scopus	1970—11/09/20	1093	1080	1134	910
Web of Science	1900 – 11/09/20	1730	1254	1820	1184
SciELO	2002 – 15/09/20	29	28	29	26
ERIC^a^	1966—15/09/20	19	2	19	2
BEI	1996—15/09/20	2	2	3	1
CDAS^a^	1927—15/09/20	13	3	13	2
ASSIA	1987—15/09/20	84	81	87	48
Open Gray^a^	1992—15/09/20	4	4	4	4
PROSPERO^a^	2011—15/09/20	1	0	2	0
Other (reference lists etc.)	-	-	-	0	0
**TOTAL ABSTRACTS**				**5675**	**3837**

ERIC: Education Resources Information Centre, ASSIA: Applied Social Sciences Index and Abstracts, CDAS: Child Development & Adolescent studies, BEI: British Education Index

*Although first established in 1996, composite nature of Cochrane library means it does not have a “start date” as other biomedical databases do

^a^includes theses, dissertations, teaching guides and other such non- peer-reviewed “grey” literature

#### Data Items

The following data points were collected using the following definitions:

##### Instrument Reference

This referred to the name of lead author and publication year of the paper.

##### Instrument Name

This referred to the instrument name and version under consideration.

##### Outcome Variable

The outcome variables of interest were executive functioning and adaptive functioning. We collected data on which outcome of interest was considered in the paper. Executive functioning was defined as “those capacities that enable a person to engage successfully in independent, purposive, self-serving behaviour, with specific executive functions consisting of initiation, planning, purposive action, self-monitoring, self- regulation, decision-making or flexibility, inhibition and volition” as reported by Stuss (Stuss, 2011). Adaptive functioning was defined as behaviours necessary for age-appropriate, independent functioning in social, communication, daily living or motor areas (Matson et al., 2009).

##### Country Settings

The desired setting was ‘developing country’ setting which was defined according to the World Bank list of lower income (LIC) (GNI per capita less than $1025), Lower-middle-Income Country (LMIC) (GNI per capita between $1026 to $3995) and upper-middle-income country list (UMIC) (GNI per capita between $3,996 TO $12,375) (Cochrane Library, 2012; World Bank Group, 2019), which were collectively referred to as “low-and-middle-income countries” in this paper. This broad approach to defining “developing country” was taken because we wanted to only exclude high-income countries, since the goal of the review was to capture tools that had been adapted for use in relatively low-resource cross-cultural settings. In that regard, we felt using as broad a definition as possible would make the findings more relevant to a lot more readers working in non-high-income country settings. Secondly, the decision to use the 2012 data was guided by the fact that we used an extremely detailed country list of low-and-middle-income countries developed by the Cochrane Library (Cochrane Library, 2012), in which rather than simply using only terms like “developing country” or “LAMIC” in the search term, the names of all 160 + non-high-income country countries (including their former names and abbreviated names- see Appendix I) could be directly entered into the search strategy. This Cochrane list was based on 2012 world bank GNI levels and was employed to be extremely thorough in our search and to improve the accuracy of the results. Having said that, when an abstract was selected using this method, the full paper was further screened to ensure that at the time the data was collected, the country was still on the World Bank List of LIC, LMIC or UMIC countries. This was done by cross-referencing data collection dates in the paper with historical data obtained from the World Bank (World Bank Group, 2019).

##### Type of Study

Data on whether the study was an adaptation or validation of an existing instrument, or a development of a new instrument was extracted, usually from the aims and objectives, methods, or results section, and documented. ‘Validation of an assessment tool’ was defined according to specific items or criteria used for reliability and validity according to the **CO**nsensus-based **S**tandards for the selection of health status **M**easurement **IN**struments) checklist items (COSMIN) guidelines (Mokkink et al., 2018; Prinsen et al., 2018). Specific items (including their taxonomy and definitions) that were included as part of validation if they were reported upon were defined as follows:

- ***Reliability:*** The extent to which scores for patients who have not changed are the same for repeated measurement under several conditions. This comprises of the following subsets:

- ⚬* Internal consistency*: The degree of the interrelatedness among the items. In other words, internal consistency is the maintenance of the same score for the same patient when different sets of items from the same instrument are used.
- ⚬* Measurement error*: The systematic and random error of a patient’s score that is not attributed to true changes in the construct to be measured.
- ⚬* Reliability*: The proportion of the total variance in the measurements which is due to ‘true’ differences between patients.

- ***Content Validity*** (**including face validity**): The degree to which the content or items of an instrument is an adequate reflection of the construct to be measured.
- ***Construct Validity***: The degree to which the scores of the instrument are consistent with hypotheses (for instance regarding internal relationships, relationships to scores of other instruments, or differences between relevant groups) based on the assumption that the instrument validly measures the construct to be measured. This will comprise of the following subsets:

- ⚬* Structural Validity*: The degree to which the scores of an instrument are an adequate reflection of the dimensionality of the construct to be measured.
- ⚬* Cross-cultural Validity*: The degree to which the performance of the items on a translated or culturally adapted instrument are an adequate reflection of the performance of the items of the original version of the instrument.
- ⚬ Hypothesis-testing: How well an expected hypothesis of how the tool is expected to behave, is fulfilled (i.e., how well the tool behaves as expected). Specific examples of hypothesis-testing assessed here were “discriminant validity” (how well the tool discriminates between expected population groups, such as autism patients versus non-patients) and “convergent validity” (how well the assessed tool converges with another similar tool in terms of their expected scores).

- ***Criterion Validity:*** The degree to which the scores of an instrument are an adequate reflection of a ‘gold standard’. For outcome measurement instruments the ‘gold standard’ is usually taken as the original full version of an instrument where a shortened version is being evaluated.
- ***Responsiveness:*** The ability of an instrument to detect change over time in the construct to be measured.
- ***Interpretability:*** Interpretability is the degree to which one can assign qualitative meaning ‐ that is, clinical or commonly understood connotations – to an instrument’s quantitative scores or change in scores. Although not a measurement property, it is an important characteristic of a measurement instrument.

Any paper that reported information on any of the above was considered as eligible for having included an eligible outcome measure. In accordance with the COSMIN guideline (Mokkink et al., 2018; Prinsen et al., 2018), “study” was defined as any individual validation conducted in any given research project or paper. For example, a given paper might report the conduct of construct validation, cross cultural validation, and structural validation of one instrument all within the same paper. This was thus reported as three studies reported within one paper.

##### Target Population

The exact age-category of children that were included.

##### Mode of Administration

Whether the instrument was a performance-based task, or an informant-based tool (i.e., self-reported or parent or proxy-based questionnaire etc.).

##### Sub-domains

The number of sub-scales or sub-domains or items of interest of the tool in question.

##### Language of Publication

Which language the paper was originally published in.

##### Sample Size

Number of participants used.

##### Demographics

Mean age and gender percentages of sample.

##### Local Settings

Whether study was predominantly set in rural or urban settings (or both).

##### Condition

Whether study was conducted among a healthy sample or clinical sample, and if so, what clinical condition.

##### Language of population

What local language-group was the study conducted among.

#### Analysis and Synthesis of Results

A synthesis of all data to summarise findings of included studies was done independently by at least 2 reviewers, compared and consensus reached. First the number of individual instruments reported on in all eligible studies found in our search were documented. Then we grouped the instruments according to the construct they measured- EF versus adaptive function. Where we encountered a systematic or scoping review, we retrieved and screened the original papers reviewed by that systematic review according to our eligibility criteria and included any that had been missed by our search for our own independent evaluation. The extracted data were then re-categorized according to each individual instrument reported and summarised. Percentage frequencies and other statistics were calculated in Microsoft Excel spreadsheets. Results were displayed using the Data Extraction Chart as shown in Table 2 below. The results are presented below in the narrative and tabular formats, and reported according to the PRISMA-ScR checklist (Tricco et al., 2018).

**Table 2:** Characteristics of Sources of Evidence

**Instrument Reference (authors, year)**	**Name and Version of Instrument**	**Outcome Measure Assessed**	**Country/ Study Sites**	Type of Study*: Development, validation	**Target Population**	**Mode of Administration**	**Sub-scales/ number of items of interest**	**Sample size**	**Age range and mean**	**Local Setting**	**Condition (clinical/ healthy)**	**Language(s) of Population**
(Bakar et al., 2011)	Behavior Rating Inventory of Executive Function BRIEF (Gioia et al., 2000)	EF	Turkey	construct validity (convergent and discriminant)	school-age children	parent report	8 sub-scales: as above for BRIEF	80: 61 ADHD^a^, 19 controls	6—11 years;	urban	ADHD^a^ and healthy	Turkish
(de Bustamante Carim et al., 2012)	Behavior Rating Inventory of Executive Function BRIEF (Gioia et al., 2000)	EF	Brazil	structural validity and internal consistency	children and adolescents	self-report, parent, and proxy (teacher) report	8 sub-scales: inhibition, flexibility, and emotional control (behavioural regulation index), initiative, working memory, planning / organization, material organization and monitoring (metacognition index)	277 parents; 282 teachers; 112 adolescents	9.8 (3.4)	urban	healthy	Portuguese
(Burkey et al., 2015)	Behavior Rating Inventory of Executive Function BRIEF (Gioia et al., 2000)	EF	Uganda	Internal consistency	school-age children	parent report	8 sub-scales: as above for BRIEF	185 children: 28 ADHD^a^, 157 healthy	8.5 years	urban	clinical (ADHD^a^) and healthy	Luganda
(Zarrabi et al., 2015)	BRIEF (Gioia et al., 2000)	EF	Iran	construct validity (discriminant)	school-age children	parent report	8 sub-scales: as above for BRIEF	60: 30 cases, 30 controls	range: 7—12 years; mean: 9yrs (1.41)	urban	ADHD^a^ and healthy	Persian
(Rincon Diaz & Rey Anacona, 2017)	BRIEF- pre-school version (Gioia et al., 2000)	EF	Colombia	adaptation, structural validity, internal consistency, test–retest reliability, construct validity (convergent)	pre-school children	parent report	5 sub-scales: inhibition, change, emotional control, working memory and planning / organization	125	range: 2—5 years; mean 4.3	urban and rural	healthy	Spanish
(Chernoff et al., 2018)	BRIEF (Gioia et al., 2000)	EF	South Africa, Malawi, Zimbabwe, Uganda	adaptation, reliability (test–retest), measurement error, convergent validity	school-age children	parent report	8 sub-scales: as above for BRIEF	603: 244 HIV, 179 HIV-exposed,180 no HIV)	range: 5—10	rural and urban	HIV, HIV exposed but healthy, no HIV exposure	Luganda, Afrikaans, Xhosa, Shona, Zulu, Chichewa, Sesotho, and Setswana
(Selvam et al., 2018)	BRIEF- pre-school version (Gioia et al., 2000)	EF	India	structural validity, internal consistency, reliability (inter rater), cross cultural validity, construct validity (discriminant)	pre-school children	parent report	5 sub-scales: inhibition, change, emotional control, working memory and planning / organization	412	2—5 years	urban	healthy	Kannada
(Amani et al., 2018)	BRIEF- teacher (Gioia et al., 2000)	EF	Iran	structural validity, internal consistency, construct validity (convergence)	school-age children	proxy (teacher) report	8 sub-scales: as above for BRIEF	360 children, 12 teachers	range: NR	urban	healthy	Persian
(Rosetti et al., 2018)	Ball Search Field Task- BSFT (Rosetti et al., 2016), BRIEF	EF	Mexico	construct validity (convergent and discriminant (age)), internal consistency (for BRIEF)	children and adolescents	interview-based task; parent report (BRIEF)	single task	106	range: 6—16; mean: 10.2 (2.7)	urban	ADHD^a^ only	Spanish
(Rosete, 2018)	Delis Kaplan Executive Function System DKEFS Children’s Color Trails Test (CCTT); Color Trails Test (CTT) (Delis et al., 2001)	EF	Thailand	Structural validity	adolescents	interview-based tasks	Design Fluency and Verbal fluency subscales of DKEFS	156	16.44 (2.04)	urban	healthy	Thai
(Pluck et al., 2019)	Tower Test of DKEFS (Delis et al., 2001)	EF: planning, working memory	Ecuador	Structural validity, internal consistency, reliability (test- retest)	children and adolescents	interview-based task	1 task	264	range 10—20, mean 15.05	urban	healthy, but some street children and foster children with controls	Spanish
(Taşcu et al., 2012)	Wisconsin Card Sorting Test WCST (Berg, 1948)	EF	Romania	construct validity (discriminant)	Mostly children and few adults	interview-based task	single task	1101	26.7	urban	clinical and healthy controls	Romanian
(Pineda et al., 2007)	bespoke battery including: WCST- abbreviated, verbal fluency test, token test, Rey-Osterrieth complex figure ROCF (Rey, 1941)	EF in ADHD^1^	Colombia	structural validity, construct validity (discriminant)	school-age children	interview-based task	3 tasks	621 total: 249 cases, 372 controls	range: 6—11,	urban	ADHD^a^ and healthy	Spanish
(Nampijja et al., 2010)	Wisconsin Card Sorting Test (WCST),Knock-Tap Game (Go/No-Go) (Luria, 1973)	EF: cognitive flexibility, planning	Uganda	adaptation, reliability (test–retest), construct validity (discriminant)	pre-school children	interview-based task	single task (each)	64	range: 4—6; mean: 5.2	rural and urban	healthy	Luganda
(Sartori et al., 2020)	Go/No-go (Luria, 1973) app version	EF: inhibition	Brazil	content, structural validity, internal consistency, cross-cultural, and construct (convergent and discriminant)	school-age children	interview-based task	4 tasks of inhibition	306: 253 healthy, 53 DCD^b^	range: 8—10 years	urban	Development Coordination Disorder (DCDb﻿), healthy	Portuguese
(Holding et al., 2018)	Rey-Osterrieth complex figure- ROCF (Rey, 1941), Go/No-Go (Luria, 1973), Shift (Holding et al., 2018)	EF: Working Memory, Inhibition, cognitive flexibility	Bangladesh, Ghana, Tanzania	adaptation, internal consistency, cross cultural validity, reliability (test–retest, inter rater), responsiveness	children	interview-based task	3 scales: each 1 item/task given multiple times	786 total: 166 Ghana, 323 Tanzania, 297 Bangladesh	range: 7—18; mean: 13	rural	healthy	Kasem and Nankam (Ghana), Swahili (Tanzania), Chatagonian (Bangladesh)
(Kashala et al., 2005)	Design Copying, Tower Test (from NEPSY^3^) (Korkman, 1998), Digit Span (backwards) (Blackburn & Benton, 1957)	EF: planning, working memory	Democratic Republic of Congo	Structural validity, construct validity (discriminant)	children	interview-based task	Design Copying, Tower Test (from NEPSYc), Digit Span (backwards)	185 (28 cases, 157 controls)	7—9.9 years, mean 8.5 years	urban	mixed: 15% clinical (ADHD^a^), 85% healthy	French; assorted African dialects
(Trevisan et al., 2017)	Children’s Executive Function Inventory CHEXI (Thorell & Nyberg, 2008)	EF in ADHD^1^	Brazil	adaptation (development), structural validity, internal consistency, construct validity (convergence or concurrent)	pre-school children	parent report/ proxy report	4 subscales: working memory, inhibition, self-regulation, and planning	408 parents and teachers	range 4—7, M = 5.51, SD = 0.59	urban	ADHD^a^ and healthy controls	Portuguese
(Thorell et al., 2020)	Teenage Executive Functioning Inventory TEXI (Thorell et al., 2020)	EF: Working memory, inhibition	Serbia	adaptation, structural validity, internal consistency, reliability (inter-rater)	adolescents	self-report, parent, and proxy (teacher) report	20 items, 2 subscales: inhibition and working memory	302 adolescents	range: 13—19; mean 16.4 (1.8)	rural and urban	healthy	Serbian
(Ruffieux et al., 2010)	Assessment Battery containing: Color Trail Test, Hand movements, Verbal Fluency, letter numbering sequence (Ruffieux et al., 2010)	EF in Sickle Cell Disease	Cameroon	Structural validity, inter rater reliability	children and adolescents	interview-based task	14 tasks: but only 5 relevant for EFs	125	range: 6—20, mean 11.4 (4.2)	urban	healthy	French, unnamed local dialects
(Injoque-Ricle et al., 2011)	The Automated Working Memory Assessment AWMA (Alloway, 2007)	EF: working Memory	Argentina	adaptation (development), internal consistency, construct validity (convergent)	school-age children	interview-based task	12 tasks: Digit Recall, Word Recall, Non-word Recall, Dot Matrix, Block Recall, Mazes Memory, Listening Recall, Counting Recall, Backward Digit Recall, Odd One Out, Mr. X Spatial Span	26 adaptation; 210 validation	range 6—11	urban	healthy	Spanish
(Wong et al., 2012)	Ballet Executive Scale BES (Wong et al., 2012)	EF in dance/ballet	Cuba	structural validity, internal consistency, concurrent validity	adolescents	self-report	5 subscales: Strategic Planning, Organization of Dance Behavior, Motivation to Dance, Impulse Control in Dance Behavior, Empathy toward Other Dancers	149	range: 11—18	urban	healthy	Spanish
(Sobeh & Spijkers, 2012)	Testbatterie zur Aufmerksamkeitsprüfung für Kinder KITAP (Zimmermann et al., 2002)	EF; Attention, inhibition, cognitive flexibility	Syria	Cross cultural validity, Construct validity (discriminant)	school-age children	performance-based task	Flexibility (The Dragon's House), Go/No-Go subscales	143	8	urban	healthy	Syrian Arabic
(Malek et al., 2013)	Stroop Colour Word Test (Stroop, 1935) Victoria Edition	EF: inhibition	Iran	construct validity (discriminant), reliability (test–retest)	adolescents	interview-based task	single task	180: 150 healthy, 30 ADHD^a^	range: 12—17 years	urban	ADHD^a^ and healthy	Persian and Turkish
(Senturk et al., 2014)	Junior Brixton Test JBT (Shallice et al., 2002)	EF	Turkey	Internal structure (structural validity), construct validity (convergent)	School-age children	interview-based task	single task	121	7.2	urban	healthy	Turkish
(Garcia-Barrera et al., 2015)	Behavioural Assessment System for Children BASC (Reynolds & Kamphaus, 1992)	EF	Colombia	internal consistency, cross cultural validity, Construct validity (discriminant)	school-age children	parent report	Not stated	848 healthy; 155 clinical	6—11 years	urban	ADHD^a^ and healthy	Spanish
(Siqueira et al., 2016)	Child Hayling Test (Burgess & Shallice, 1997) Brazilian version	EF: inhibition, cognitive flexibility	Brazil	adaptation (development)	school-age children	interview-based task	24 items	139	range: 6—12	urban	healthy	Portuguese
(Sallum et al., 2017)	Self-Ordered Pointing Task SOPT (Petrides & Milner, 1982)	EF: working Memory	Brazil	construct validity (discriminant and convergent), ecological validity	pre-school children	interview-based task	single task	248	range: 3—5	urban	healthy	Portuguese
(Richard’s et al., 2017)	Conjunction Visual Search (CVS) task of tareas de autorregulaCión Cognitiva TAC battery (Richard’s et al., 2017)	EF: inhibition	Argentina	development (but of computer-based task), convergent validity	school-age children	interview-based, computer administered task	1 task: 120 trials	41	range: 6—11, mean: 8.49; standard deviation: 1.47	urban	healthy	Spanish
(Yang et al., 2018)	Dysexecutive Questionnaire DEX (Wilson et al., 1996)	EF	China	structural validity	adolescents	self-report	20 items	1586	mean: 18.9 (5.4)	urban	healthy	Mandarin
(Barreto et al., 2018)	Neuropsychological Assessment of Executive Functions (Spanish acronym: ENFEN) (Portellano et al., 2009)	EF	Colombia	structural validity, construct validity (convergent and discriminant)	school-age children	interview-based task	Not stated	367 children	range: 6—12; mean 8.9 (1.8)	rural and urban	healthy	Spanish
(Du et al., 2018)	Symptoms and Functional Impairment Rating Scale SFIRS (Du et al., 2018)	EF in ADHD^1^: working memory, planning, time management, self-monitoring, and emotional control	China	development, structural validity, internal consistency, reliability (test- retest), construct validity	school-age children	parent report	6 sub-scales: hyperactivity-impulsivity, self-control, inattention, self-management, school performance, social interaction	20 development; 412 validation	range: 6—12, 8.7 (1.42)	urban	ADHD^a^ and healthy	Mandarin
(Richard’s et al., 2018)	Tareas de Autorregulación Cognitiva Battery (TAC)	EF: inhibition, working memory and cognitive flexibility	Argentina	structural validity	children	interview-based task	3 sub-scales used: perceptual inhibition task, 4 WM tasks; 1 cognitive flexibility task	103 children	range: 9—12 years; M = 10.84 SD = 0.88	urban	healthy	Spanish
(Green et al., 2019)	The Cambridge Neuropsychological Test Automated. Battery CANTAB (Huppert et al., 1995)	EF: working memory, inhibition, and attention shifting	Mexico	construct validity (discriminant)	children and adolescents	interview-based task, computer based	6 sub-tests (out of 23): stockings of Cambridge, delayed matching sample, extra dimensional shift, rapid visual information processing, stop signal task and match to sample visual search task	826 children	range 5—15; mean 9.27 (2.6)	urban	healthy	Spanish
(Ford et al., 2019)	Rapid Assessment of Cognitive and Emotional Regulation RACER (Ford et al., 2019)	EF	Lebanon, Niger	cross cultural validity	children	interview-based, computer administered task	2: inhibition; working memory	2725; 866—Niger, 1859 Lebanon	Lebanon: 9.2 (2.3); Niger: 9.2 (1.4)	urban	healthy	French; Arabic
(Korzeniowski & Ison, 2019)	Executive Function Scale for Children EFS (Korzeniowski & Ison, 2019)	EF	Argentina	content validity, structural validity, internal consistency, reliability (test–retest)	school-age children	parent report	6 sub-scales: attention control, inhibitory control, metacognition, organization, planning and cognitive flexibility	307	7.7 (1.07)	urban	healthy	Spanish
(Willoughby et al., 2019)	EF Touch (computer-based battery) (Willoughby et al., 2019)	EF: inhibition, cognitive flexibility and working memory	Kenya	construct validity (discriminant)	pre-school children	computer-based, interview administered task	5 tasks/subscales: Go/no-go, variant of Stroop, Spatial conflict arrows, Pick the picture, Something's the same	193	range: 3—6yrs	urban	healthy	Kiswahili, English
(Gonen et al., 2019)	Preschool Self-Regulation Assessment. PRSA (Smith-Donald et al., 2007)	EF in pre-schoolers: self-regulation	Turkey, USA	cross cultural validity, reliability	pre-school children	interview-based task	3 tasks: The Balance Beam, Tower Task and Pencil Tap tasks	Turkey: 471 children + caregivers; USA: 286 children + caregivers	Turkey: 5.23yrs (2.83 to 5.92); US 4.12 yrs (2.97 to 5.07)	urban	healthy	Turkish, English
(Xu et al., 2020)	computerized version of: Stop Signal task (Xu et al., 2020), Figure Matching task, Spatial Span task; Tower of Hanoi task (Simon, 1975)	EF	China, Hong Kong and USA	cross cultural validity	school-age children and adolescents	self-administered computer-based tasks	4 tasks each measuring: inhibition, cognitive flexibility, working memory, planning	China: 453; Hong Kong 371; UK 487	HK: 12.2yrs, UK: 11.9yrs; China: 11.93yrs	urban and rural	healthy	Mandarin, Cantonese, English
(Mashhadi et al., 2020)	Barkley Deficits in Executive Functioning Scale–Children and Adolescents BDEFS-CA (Barkley, 2012)	EF	Iran	structural validity, internal consistency, reliability (test–retest), construct validity (convergent and discriminant)	children and adolescents	parent report/self-report	5 subscales: Self- Management to Time, Self-Organization/Problem Solving, Self-Restraint, Self-Motivation and Self-Regulation of Emotion	2295	range: 6—18; mean: 11.6 (3.34)	rural and urban	mostly healthy, 4.7% clinical	Persian
(Arruda et al., 2020)	EFICA (Executive Function Inventory for Children and Adolescents)- parent and teacher versions (Arruda et al., 2020)	EF	Brazil	structural validity, internal consistency, reliability, construct validity (discriminant)	children and adolescents	parent report/ proxy (teacher) report	Not stated	3284 healthy, 165 ADHD^a^	range 5—18, mean 8.2 (2.0)	rural and urban	ADHD^a^ and healthy	Portuguese
(Tombokan-Runtukahu & Nitko, 1992)	Indonesian- Vineland Adaptive Behaviour Scales VABS (Sparrow et al., 1984, 2005)	adaptive functioning	Indonesia	adaptation (development), internal consistency, reliability (intra-rater and inter rater), cross cultural validity, Construct validity (discriminant)	children	parent report	4 domains: Communication, Daily Living Skills, Socialization, and Maladaptive Behavior	86: 43 ID^d^, 43 healthy	range: 6- 18; mean: 11.8 (2.9)	semi-urban	Intellectual disability (IDd), healthy	Indonesian
(Goldberg et al., 2009)	VABS- Vietnam version	adaptive functioning	Vietnam	adaptation, structural validity, internal consistency, construct validity (discriminant)	pre-school children	parent report	4 domains: as above for VABS	120 healthy, 31 ID^d^ children	range: 3—6yrs; mean 4.9 (1.1)	urban	intellectual disability and healthy	Vietnamese
(Selvam et al., 2016)	VABS II- Indian Version	adaptive functioning	India	cross cultural validity	pre-school children	parent report	4 domains: as above for VABS	412	2—6 years,	urban	healthy	Kannada
(Amini et al., 2016)	Iranian Children's Participation Assessment Scale CPAS (Amini et al., 2016)	Adaptive functioning	Iran	Scale development	Children	parent report/self-report	8 subscales: ADL, Instrumental ADL, Play, Leisure, Social Participation, education, Work, Rest/Sleep	40 kids, 21 parents	11.5 (2.8); range 6—18 years	urban	healthy	Persian
(Amini et al., 2017)	Children's Participation Assessment Scale (CPAS)- parent version	adaptive functioning	Iran	structural validity, reliability (test–retest), construct validity (convergent)	school-age children	parent- report	8 subscales: ADL, Instrumental ADL, Play, Leisure, Social Participation, education, Work, Rest/Sleep	700 parents	range: 6—12; mean 9.45 (1.76)	urban	healthy	Persian
(Munir et al., 1999)	Independent Behaviour Assessment Scale IBAS (Munir et al., 1999)	adaptive functioning	Bangladesh	reliability, internal consistency, construct validity (discriminant)	pre-school and school-age children	parent report, interview-based tasks	4 subscales: Motor skills, Socialisation skills, Communication skills, Daily living skills	1404 healthy, 222 clinical	range: 2—9,	rural and urban	healthy and clinical	Bengali
(Holding & Kitsao-Wekulo, 2009)	Participation in Activities of Daily Living PADL (Holding & Kitsao-Wekulo, 2009)	adaptive functioning	Kenya	development, internal consistency, construct validity (discriminant)	children	interview-based questionnaire	2 subscales: participation and limitations to participation	study development: 92, validation: 116	range: 6—18; mean: 11.7 (2.9)	rural	assorted chronic conditions (Sickle Cell, DM^e^, etc.); acute encephalopathy; healthy controls	Kiswahili
(Tol et al., 2011)	Child Function Impairment Rating Scale CFIRS (Tol et al., 2011)	adaptive functioning	Indonesia	development, Structural Validity, internal consistency, reliability (test- retest, inter rater), construct validity (convergent and discriminant)	children	FGD, diaries, interview-administered questionnaire	1 unidimensional scale with 4 factors; 11 items,	development: 53, validation: 403 children, 385 parents	mean: 9.9 (1.21)	rural	healthy but exposed to political violence	Indonesian
(Malkawi et al., 2015)	Arabic Preschool Activity Card Sort A-PACS (Berg & LaVesser, 2006)	adaptive functioning, ADL	Jordan	development	school-age children	parent report	7 domains: self-care, community mobility, high physical demand leisure and low physical demand leisure, social inter- action, domestic and education	115 caregivers	4.8	rural and urban	healthy	Arabic
(Amini et al., 2017)	Iranian- Child Participation Questionnaire I-CPQ (Rosenberg et al., 2010)	adaptive functioning	Iran	Structural validity, internal consistency, reliability, construct validity (convergent)	pre-school children	parent report	6 subscales: self-care, home participation, play, leisure, social participation, and educational environment	120	range: 4—6; mean: 5.2	urban	cerebral palsy	Persian

*Studies as defined according to COSMIN guidelines as any individual validation conducted in any given research project/paper. Thus, some individual papers reported multiple validation “studies” within that single paper

^a^ADHD- Attention Deficit/Hyperactivity Disorder

^b^DCD- Development Coordination Disorder

^c^NEPSY- A Developmental NEuroPSYchological Assessment

^d^ID- Intellectual Disability

^e^DM- Diabetes Mellitus

## Results

### Selection of Sources of Evidence

Table 1 summarises the results of the initial search in each individual data source, along with the dates of coverage of the search in each database. The databases with the most hits given the search criteria were Web of Science, PsychINFO, Scopus and MEDLINE, which was expected given their respective scopes of subject matter as discussed above (see *Information Sources*).

After an automatic de-duplication using Mendeley, the scoping review identified 3837 potentially eligible abstracts for manual screening. Of these a further 72 were excluded for being duplicates (missed by the automatic de-duplication), 3091 were excluded for either not reporting any psychometric data (i.e., not being development or validation or adaptation studies) or not being about EF or adaptive functions at all, while 104 were studies focused wholly or mostly on adult populations. 570 full articles were thus further screened for eligibility, with 519 being excluded for such reasons as failure to translate into English (23 papers), country settings being predominantly high-income (220 papers), and the study being about an adaptive function tool being used in a non-brain pathology or physical injury context such as limb amputation (28 papers), among other reasons (see Fig. 1 for summary). Ultimately, 51 full papers were found to be eligible for full data extraction and review.

### PRISMA Flowchart for Study Selection

#### Characteristics and Results of Individual Sources of Evidence

Table 2 presents the characteristics of the data extracted from all eligible papers (sources of evidence) in the data extraction chart. As can be seen from the ‘Type of Study” column of this table, several papers reported multiple “studies” in a single paper, where “study” was defined as an individual validation as recommended by the COSMIN guideline. For example, in Senturk et al. (2014) paper on the Junior Brixton Test, they reported on two studies- both structural validity and construct validity- for the JBT. By this count, a total of 163 studies were reported in 51 papers. When disaggregated into individual studies, the most frequently conducted type of study (in descending order) were structural validity and construct validity or hypothesis testing studies at 38 studies each (23.3% each of total individual studies), followed in order by internal consistency studies at 27 (16.6%), reliability studies at 23 (14.1%), cross cultural validity studies at 14 (8.6%), adaptation or content validity studies at 13 (7.9%), instrument development at 6 (3.7%), responsiveness 3 (1.8%) and measurement error 1 (0.6%). Tables 3 and 4 in Appendix III shows the breakdown of these by EF individual instruments and adaptive function individual instruments, and are reported on in ‘Synthesis of Results’ below. Informant-based instruments (either by self, parents, or another proxy) were slightly more frequent at 29 instances (53.7%) compared to performance-based measures at 25 instances (46.3%). The top 3 low-and-middle-income countries in which these validation studies were conducted were Iran (7 papers), Brazil (6 papers) and Colombia and Argentina (4 papers each). But when categorized in terms of world regions (i.e., regions with broadly similar cultural or linguistic environments), the top performing regions with the most papers reporting validation studies from them were Latin America (Central and South America) with 17 papers (30.4% of instances), Sub-Saharan Africa 12 papers (21.4%), the Middle East 10 papers (17.9%) and South-East Asia (including the Indian sub-continent) with 8 papers (14.3%). Most studies conducted involved urban populations at 46 instances (76.7%), and exclusively healthy populations at 31 instances (60.8%) as opposed to clinical populations (with healthy controls) at 20 instances.

#### Synthesis of Results

**Fig. 2:**
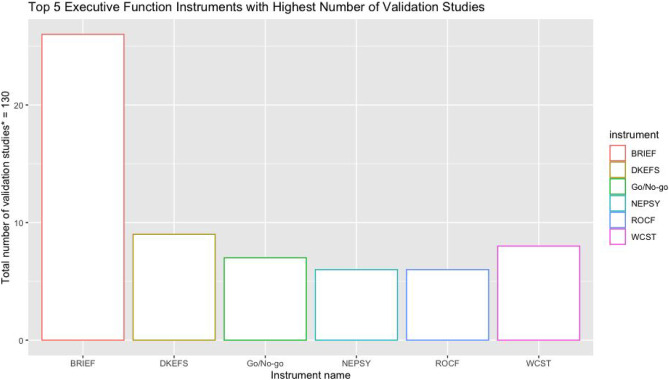
Top performing EF instruments in terms of total number of validation studies. *Studies is defined according to COSMIN guidelines as any individual validation conducted in any given research project/paper. Thus, some individual papers reported multiple validation “studies” within that single paper

**Fig. 3:**
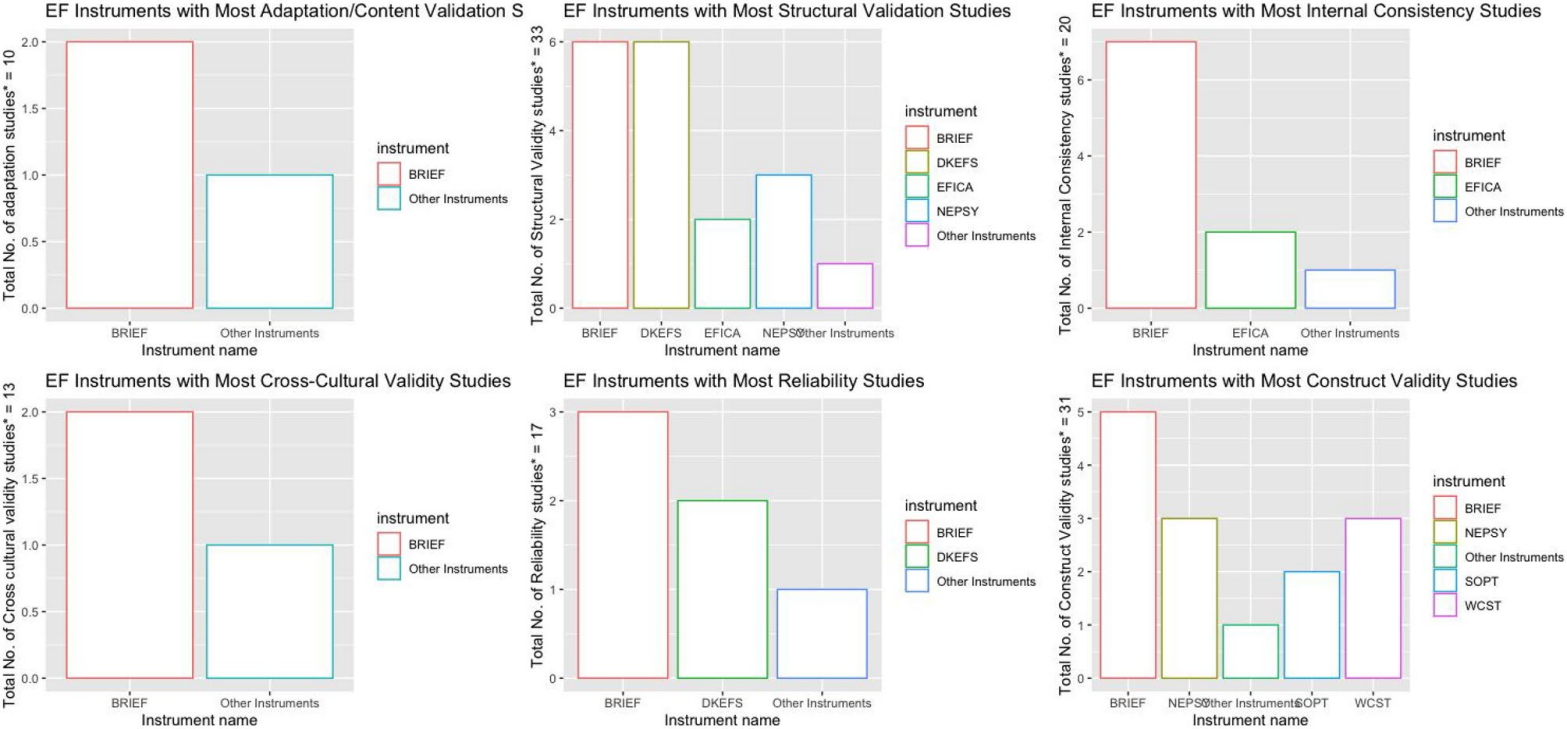
Break-down of most validated EF instruments in terms of individual types of Validation studies. *Studies as defined according to COSMIN guidelines as any individual validation conducted in any given research project/paper. Thus, some individual papers reported multiple validation “studies” within that single paper

In this sub-section, the results are disaggregated according to the individual instruments and reported on. In this scoping review, 40 unique tools, including 49 version or variants, were identified as having been either developed or adapted or validated for use among children in low-and-middle-income countries from the 51 papers reviewed. A total of 130 individual studies were done for the EF instruments reported on in this review (see Table 3 in Appendix III). Figure 2 shows the top 5 EF instruments that reported any validation study. BRIEF (Gioia et al., 2000), in all its various versions, was by far the most validated instrument in terms of numbers of reported studies with 26 validation studies in total (20%), followed by DKEFS (Delis et al., 2001) (9 studies- 6.9%), WCST (Berg, 1948) (8 studies- 6.2%), Go/No-go (Luria, 1973) (7 studies- 5.4%)) and NEPSY (Korkman, 1998), and ROCF (Rey, 1941) (6 studies each- 4.6%). This is a remarkable performance for the BRIEF as it was developed only in the last 20 years compared to the legacy tests WCST and Go/No-go which have been existence for over 70 years, hence it is not surprising that several studies have been done on those tests. In Fig. 3, these results were broken down by types of validation studies and again summarised by top-performing instruments (Table 3 in Appendix III shows the full results of the frequencies of specific types of validation studies done for all individual Executive Function instruments). In this figure, only instruments with 2 or more validation studies were individually named, with all instruments that had only one validation study in any particular validation study type being grouped under “other instruments”. When these results were thus broken-down interesting results emerged. Firstly, the BRIEF maintained its status as the most validated instrument regardless of the type of validation- from adaptation studies, through cross-cultural validity studies to construct validity studies. This perhaps speaks to the popularity of the BRIEF even in low-and-middle-income countries. Only the WCST and Go/No-go tests showed a similar consistency of validation with at least 1 validation study in almost all the category types of validation studies although in the graph below this is subsumed under the “other instruments” category (see Table 3 in Appendix III). Apart from BRIEFs and Go/No-go, performance for the other instruments were mostly not consistent across the various types of validation studies. NEPSY for example had all its validation studies in only two categories, including structural validity (3) and construct validity studies (3), while DKEFS featured mostly under structural validity studies (6), reliability studies (2) and Construct validity studies (1) (see Table 3 Appendix III).

**Fig. 4:**
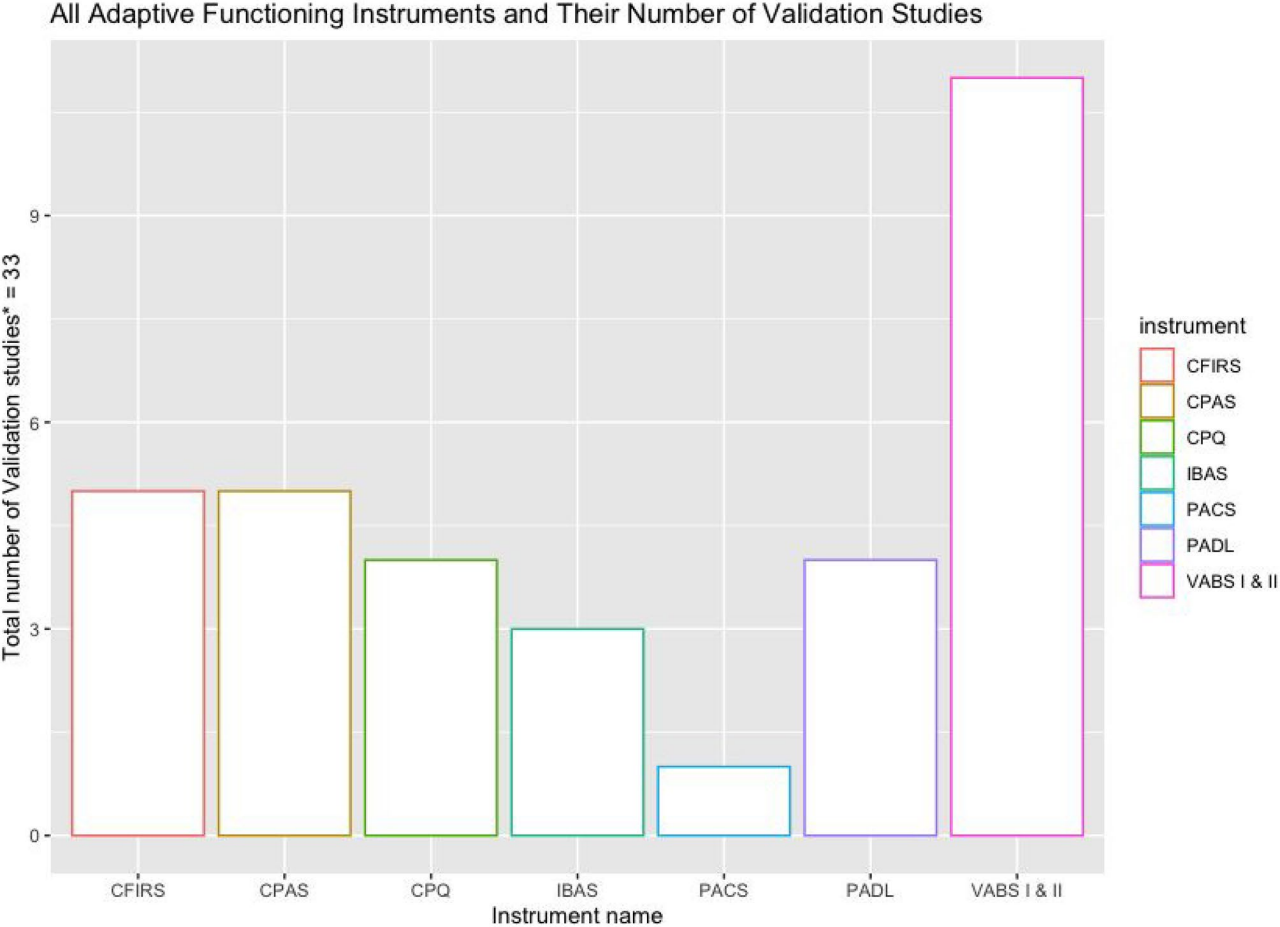
Top performing adaptive functioning instruments in terms of total number of validation studies. *Studies as defined according to COSMIN guidelines as any individual validation conducted in any given research project/paper. Thus, some individual papers reported multiple validation “studies” within that single paper. CFIRS: Child Function Impairment Rating Scale. CPAS: Children’s Participation Assessment Scale. CPQ: Child Participation Questionnaire. IBAS: Independent Behaviour Assessment Scale. PACS: Preschool Activity Card Sort. PADL: Participation in Activities of Daily Living. VABS: Vineland Adaptive Behaviour Scale

Figure 4 is a graph of the most validated adaptive functioning instruments by number of validation studies conducted among children in low-and-middle-income countries. For a full display of results for all validation study types for Adaptive Function instruments used following brain pathology, see Table 4 in Appendix II. A total of 33 individual studies were done for the adaptive function instruments reported in this review. By far, the VABS (in all its various iterations or editions) (Sparrow et al., 1984, 2005) is the most validated instrument for adaptive functioning following brain pathology in children in low-and-middle-income countries with 11 validation studies (33.3%), distantly followed by the CFIRS (Tol et al., 2011) an newly developed tool from Indonesia and the CPAS (Amini et al., 2016) with 5 studies (15.2%) each.

## Discussion

This scoping review was carried out to find out which instruments for assessing executive function and adaptive functioning among children had been validated in low-and-middle-income countries. It also sought to establish which of these instruments stood out in terms of the number and variety of validation studies conducted. This is the first such scoping review in the context of low-and-middle-income countries to the best of the authors’ knowledge.

### The Most Validated Instruments

Judging solely by number of validation studies, the BRIEF (Gioia et al., 2000) appears to be the most validated instrument for executive functions among children aged 6 – 18 years in low-and-middle-income countries, while the VABS (Sparrow et al., 1984, 2005) is the most validated for adaptive functioning in a similar context. This finding is corroborated by a similar scoping review of EF instruments used around the world among adolescents where BRIEF was among the top 3 instruments (Nyongesa et al., 2019).

However, the variety of types of validation studies done in non-high-income country was quite limited. None of the instruments found in this present paper had a validation study in all major categories of validation considered (see Table 3 Appendix III). In fact, of the EF tools, apart from the BRIEF (Gioia et al., 2000), WCST (Berg, 1948) and Go/No-go (Luria, 1973) tests, most of the others had validation studies in only 2 to 3 out of the 9 categories of validation studies. Indeed, just 3 types of validation studies- structural validity, construct validity and internal consistency- accounted for a disproportionate 63% of all the validation studies. This showed that validation studies in low-and-middle-income countries was generally skewed towards a few types of studies, highlighting a paucity of studies examining such validation categories as measurement error, responsiveness, content validation and cross-cultural validation. Specific to content validation, unfortunately, relatively few studies focused on adapting or content-validating an existing western-derived instrument (7.9%) or developing a new instrument de novo (3.7%) for the low-and-middle-income country context. In these cases, the implicit but potentially erroneous assumption is that the content of these tools will already be valid in these low-and-middle-income countries (hence making content validation unnecessary), with the focus thus being on other forms of validation (construct, structural etc.). This is noteworthy because one would expect that validating the content of a foreign tool in a new context would be one of the first and most important adaptations to be done before any others are considered (Terwee et al., 2018). Therefore, while many validation studies for EF and adaptive function instruments have been conducted in low-and-middle-income country contexts, a much wider variety of studies is needed, particularly content validity studies.

Considering the distribution of validation studies in low-and-middle-income countries by world regions, the highest performing regions were Latin America (30.4%), sub-Saharan Africa (21.4%) and the Middle East (17.9%). This is a telling finding because of the implications it has for imposing language limitations in the methodology of such scoping reviews, even if it is for understandable reasons of resource constraints. Apparently, work is progressing in countries like Brazil, Argentina and Iran (the 3 top performing low-and-middle-income countries by number of validation papers). These are non-English speaking countries and are likely to be over-looked by English-based scoping or systematic reviews sadly. Thus, even though in this present study we were also unable to translate and thus include several non-English language papers (see Appendix II for list of such potentially eligible papers that were not included for lack of translation), we did not impose any language restrictions to our search a priori and made the effort to translate as many as we could. Given the performance of these non-English countries or regions, one wonders how skewed the results might have been had such language restrictions been imposed.

Another interesting observation was that Informant-based instruments (either by self, parents or another proxy) were slightly more frequent at 53.7% compared to performance-based measures at 46.3%, which was in contrast to the findings by Nyongesa and colleagues (Nyongesa et al., 2019). This might be because of differences in focus and methodology of the two papers. We focused exclusively on low-and-middle-income countries but also looked broadly at children and adolescents aged 5 – 18 years, while they focused more broadly on all countries (including high-income countries) but looked specifically at adolescents aged 13 – 17 years. In terms of methodology, we looked at 14 databases and imposed no language or date restrictions, while they searched 3 databases and focused on the last 15 years only and on papers published only in English. Evidently when the search is broadened to include high-income countries, performance-based measures dominate while the reverse is true when the search is limited to low-and-middle-income countries. This may possibly be explained by positing that in studies from high-income countries, performance-based measures are most used and largely in experimental, theoretically driven studies that tend to be conducted in controlled environments where performance-based measures work best. In contrast, in studies from low-and-middle-income countries, informant-based questionnaires tend to be preferred, and mostly used in clinical-based studies because they are probably easier to administer and score in a clinical context, and they tend to have better ecological validity (Gioia et al., 2010; Nyongesa et al., 2019) and thus work best in the clinical context.

Further, most validation studies in low-and-middle-income countries took place in urban centres (76.7%). This is somewhat concerning because of the major socioeconomic disparities and standard of living that exist in low-and-middle-income countries between urban and rural populations. The effects of socioeconomic status (SES) on performance in executive function testing has been well documented in South Africa, Australia (Howard et al., 2020) and the United States (Raver et al., 2013). Given that the majority of people in low-and-middle-income countries still live in poverty in rural areas, a lot more validation studies in low-and-middle-income countries involving rural populations are needed to better reflect the realities on the ground in those countries.

#### The Cross-Cultural Conundrum

There were relatively few cross-cultural validations (only 8.6% of studies) across board. This is an important observation because this review focused on low-and-middle-income countries in which most of these instruments were not the original countries or cultures of development. Even the BRIEF (Gioia et al., 2000), the instrument with the most validation studies conducted in low-and-middle-income countries had only 2 cross-cultural validation studies done, with only 11 other EF instruments (out of the 40 reviewed) each having a single cross-cultural validation study done. One would have expected more of such cross-cultural studies to perform robust cross-cultural validation of the instruments used. Lev Vygotsky was among the first to warn about the dangers of simply assuming that the concept implied in an item on any measurement instrument would automatically carry forward to another culture and be understood in the same way when that instrument is used in the recipient culture (Vygotsky, 1986 English translation). He warned that if the target audience did not truly hold the same concept as the host audience in the use of a particular set of words (even if they were “translated” words), they were likely to mishandle the item and therefore produce results that were not truly reflective of whatever concept was being assessed (Vygotsky, 1986 English translation). The implication of these is that an assumption could not simply be made by a cross-cultural researcher ab initio that any behaviours (or the concepts underlying them) would be developed at all or developed in the same way as within the culture of origin of the researcher (Vygotsky, 1986).

In recent times, this very point has been reinforced by cross-cultural researchers using modern psychometric techniques by demonstrating that assessment of item bias and measurement invariance of any tool used in a cross-cultural context is a crucial step in the comparison of any two groups, for meaningful conclusions to be made (Fischer & Karl, 2019; Gannotti & Handwerker, 2002). The purpose of evaluating invariance is to confirm whether the responses of different populations on each item differ by more than chance, or put in other words, whether the properties of an instrument are the same in two different groups. Lack of invariance in two groups means there are systematic differences in the way the two groups answer the same questions (for example, the lack of conceptual equivalence that Vygotsky alluded to). The important implication is that if the performance of an instrument is not comparable across two groups (for example, if the factor structure is quite different), one cannot compare the two groups on the construct that is being measured (e.g., compare their mean scores or correlations), and cannot thus draw conclusions that any perceived differences between the two groups are real differences, rather than them simply being an artefact of the fact that the instrument being used behave differently in the two groups. This therefore highlights the utmost importance of cross-cultural validation studies in low-and-middle-income countries for any western-derived assessment tool, and thus belies the unfortunate lack of these studies demonstrated by this scoping review.

### Strengths and Limitations of this Study

This scoping review was conducted following the rigour of the highly recommended PRISMA- ScR guideline (Tricco et al., 2018), ensuring that it was conducted to the highest of methodological standards and can be easily replicated by other researchers. We did not impose any date restrictions and thus went as far back in time as possible for publications available on the databases. Further, the search strategy used was a very thorough one which included listing by name (including name spelling variants) all low-and-middle-income countries in the search strategy, rather than just relying on terms like “developing country”, and so on. We also searched an exceptionally large number of databases (14 in all) and specifically searched the grey literature and in databases tailored towards low-and-middle-income countries. We also endeavoured to include as many non-English papers as we could translate in the study.

However, a significant limitation was that we failed to translate or obtain up to 27 papers that might have been eligible judging solely from their abstracts. However, judging from the eligibility rate after review of full papers of 8.9%, it can be projected that only about 2–3 eligible full papers (i.e., 8.9% of the 27 papers) would probably have been truly eligible of this list but were missed. This would probably not have significantly changed the overall conclusions made in this paper. We have however listed these potentially missed publications in Appendix II for the sake of transparency. We also did not do a robust risk of bias assessment of the methodology and results of the reported validation studies, which would have aided more definitive conclusions to be drawn. But as mentioned, this was outside the scope of this study.

## Conclusion

Quite a number of validation studies have been published on EF and adaptive function assessment tools among children and adolescents in low-and-middle-income countries, however these are woefully inadequate to cover the scope of validations out there. Particularly concerning is the lack of adaptation, content validity and cross-cultural validity studies for western-derived instruments being used in low-and-middle-income countries, as well as studies on development of instruments purposely for low-and-middle-income countries. EF and adaptive function tools that have either been adapted or developed for low-and-middle-income countries are therefore lacking and much needed. The quality of these validation studies though is outside of the scope of this scoping review paper and will be better explored in a subsequent systematic review paper.

## Supplementary Information

Below is the link to the electronic supplementary material.

Supplementary file1 (DOCX 84 KB)

Supplementary file2 (DOCX 36 KB)
