# Supplementary material for: Assessment Tools for Executive Function and Adaptive Function Following Brain Pathology Among Children in Developing Country Contexts: a Scoping Review of Current Tools
Source: Neuropsychol Rev. 2021 Dec 6;32(3):459–82. doi: 10.1007/s11065-021-09529-w (PMC9381467; doi:10.1007/s11065-021-09529-w)
Supplement: Supplementary file 2 — Supplementary file2 (DOCX 36 KB) [file 11065_2021_9529_MOESM2_ESM.docx]

# Assessment Tools for Executive Function Adaptive Function Following Brain Pathology Among Children in Developing Country Contexts: A Scoping Review of Current Tools

## APPENDICES

APPENDIX I: Search Strategy for MEDLINE

1. "executive function*".ti,ab.
2. “executive dysfunction*”.ti,ab.
3. “dysexecutive syndrome”.ti,ab.
4. "frontal lobe* function*".ti,ab.
5. "frontal lobe* dysfunction*".ti,ab.
6. "function* of the frontal lobe*".ti,ab.
7. “frontal lobe* syndrome”.ti,ab.
8. "frontal lobe* damage".ti,ab.
9. “prefrontal cortical damage”.ti,ab.
10. “prefrontal damage”.ti,ab.
11. “prefrontal cortical dysfunction*”.ti,ab.
12. “prefrontal dysfunction*”.ti,ab.
13. “prefrontal cortical function*”.ti,ab.
14. “prefrontal function*”.ti,ab.
15. “adaptive function*”.ti,ab.
16. “adaptive function* impair*”.ti,ab.
17. “activities of daily living”/
18. “daily living”.ti,ab.
19. “ADL”.ti,ab.
20. or/1-14
21. or/15-19
22. “children”.ti,ab.
23. 21 and 22
24. “valid*".ti,ab.
25. “reliab*”.ti,ab.
26. “unreliab*”.ti,ab.
27. standard*”.ti,ab.
28. “norm*”*".ti,ab.
29. “reproducibl*”.ti,ab.
30. “replica*”.ti,ab.
31. “coefficient of variation”.ti,ab.
32. “internal consistency”.ti,ab.
33. “responsive*”.ti,ab.
34. “assessment tool*”.ti,ab.
35. “assessment instrument*”.ti,ab.
36. “outcome assessment”.ti,ab.
37. “outcome measure*”.ti,ab.
38. “clinimetr*”.ti,ab.
39. “clinometr*”.ti,ab.
40. “cross-cultural”.ti,ab.
41. "reproducibility of results"/
42. "sensitivity and specificity"/
43. behavior rating scale/
44. neuropsychological tests/
45. psychometrics/
46. discriminant analysis/
47. Or/24-46
48. Developing Countr***.sh,kf.**
49. (low adj3 middle adj3 countr*)**.ti,ab.**
50. (lmic or lmics or third world or lami countr*)**.ti,ab.**
51. transitional countr***.ti,ab.**
52. ((developing or less* developed or under developed or underdeveloped or middle income or low* income or underserved or under served or deprived or poor*) adj (countr* or nation? or population? or world))**.ti,ab.**
53. ((developing or less* developed or under developed or underdeveloped or middle income or low* income) adj (economy or economies))**.ti,ab.**
54. (low* adj (gdp or gnp or gross domestic or gross national))**.ti,ab.**
55. (Africa or Asia or Caribbean or West Indies or South America or Latin America or Central America)**.hw,kf,ti,ab,cp.**
56. (Afghanistan or Albania or Algeria or Angola or Antigua or Barbuda or Argentina or Armenia or Armenian or Aruba or Azerbaijan or Bahrain or Bangladesh or Barbados or Benin or Byelarus or Byelorussian or Belarus or Belorussian or Belorussia or Belize or Bhutan or Bolivia or Bosnia or Herzegovina or Hercegovina or Botswana or Brasil or Brazil or Bulgaria or Burkina Faso or Burkina Fasso or Upper Volta or Burundi or Urundi or Cambodia or Khmer Republic or Kampuchea or Cameroon or Cameroons or Cameron or Camerons or Cape Verde or Central African Republic or Chad or Chile or China or Colombia or Comoros or Comoro Islands or Comores or Mayotte or Congo or Zaire or Costa Rica or Cote d'Ivoire or Ivory Coast or Croatia or Cuba or Cyprus or Czechoslovakia or Czech Republic or Slovakia or Slovak Republic or Djibouti or French Somaliland or Dominica or Dominican Republic or East Timor or East Timur or Timor Leste or Ecuador or Egypt or United Arab Republic or El Salvador or Eritrea or Estonia or Ethiopia or Fiji or Gabon or Gabonese Republic or Gambia or Gaza or Georgia Republic or Georgian Republic or Ghana or Gold Coast or Greece or Grenada or Guatemala or Guinea or Guam or Guiana or Guyana or Haiti or Honduras or Hungary or India or Maldives or Indonesia or Iran or Iraq or Isle of Man or Jamaica or Jordan or Kazakhstan or Kazakh or Kenya or Kiribati or Korea or Kosovo or Kyrgyzstan or Kirghizia or Kyrgyz Republic or Kirghiz or Kirgizstan or Lao PDR or Laos or Latvia or Lebanon or Lesotho or Basutoland or Liberia or Libya or Lithuania or Macedonia or Madagascar or Malagasy Republic or Malaysia or Malaya or Malay or Sabah or Sarawak or Malawi or Nyasaland or Mali or Malta or Marshall Islands or Mauritania or Mauritius or Agalega Islands or Mexico or Micronesia or Middle East or Moldova or Moldovia or Moldovian or Mongolia or Montenegro or Morocco or Ifni or Mozambique or Myanmar or Myanma or Burma or Namibia or Nepal or Netherlands Antilles or New Caledonia or Nicaragua or Niger or Nigeria or Northern Mariana Islands or Oman or Muscat or Pakistan or Palau or Palestine or Panama or Paraguay or Peru or Philippines or Philipines or Phillipines or Phillippines or Poland or Portugal or Puerto Rico or Romania or Rumania or Roumania or Russia or Russian or Rwanda or Ruanda or Saint Kitts or St Kitts or Nevis or Saint Lucia or St Lucia or Saint Vincent or St Vincent or Grenadines or Samoa or Samoan Islands or Navigator Island or Navigator Islands or Sao Tome or Saudi Arabia or Senegal or Serbia or Montenegro or Seychelles or Sierra Leone or Slovenia or Sri Lanka or Ceylon or Solomon Islands or Somalia or South Africa or Sudan or Suriname or Surinam or Swaziland or Syria or Tajikistan or Tadzhikistan or Tadjikistan or Tadzhik or Tanzania or Thailand or Togo or Togolese Republic or Tonga or Trinidad or Tobago or Tunisia or Turkey or Turkmenistan or Turkmen or Uganda or Ukraine or Uruguay or USSR or Soviet Union or Union of Soviet Socialist Republics or Uzbekistan or Uzbek or Vanuatu or New Hebrides or Venezuela or Vietnam or Viet Nam or West Bank or Yemen or Yugoslavia or Zambia or Zimbabwe or Rhodesia)**.hw,kf,ti,ab,cp.**
57. Or/48-57
58. 20 and 23 and 47 and 57

NB: for search items 48 – 56, a special search filter for ‘developing countries’ authored by the Cochrane Library (Cochrane Library, 2012) was used verbatim.

APPENDIX II: Untranslated and/or Unavailable but potentially Eligible Non-English Articles

1. Zhang Q. 2003 Development of Adaptive Skill Rating Scale for school age children: *only Chinese PDF available*
2. Yao S. 1999 Development of the Adaptive Skill Rating Scale for Young Children (ASRSYC): *online version not available*
3. Rosselli-Cock M. 2004 Neuropsychological assessment of children: A test battery for children between 5 and 16 years of age. A Colombian normative study: *online version not available*
4. Pereira A. 2018 Executive functions in childhood: Assessment and preliminary normative data for Portuguese preschoolers: *study conducted in Brazil but online pdf version not available*
5. Du Q. 2010 Reliability and validity of the Behavior Rating Inventory of Executive Function-Adult Version Self-Report Form in China: *pdf version not available online*
6. Singh S. 2019 A comparative study of vineland adaptive behavior scale ii and vineland social maturity scale on children and adolescents with intellectual disability: *online version not readily available*
7. Pawlowski J. 2014 Evidences of construct validity of the NEUPSILIN using confirmatory factorial analysis: *only Portuguese pdf available, was unable to get translator*
8. Mashhadi A. 2014 Psychometric properties of the Behavior Rating Inventory of Executive Functioning-Preschool Version (Teacher Form): *only Persian version of pdf available, hence was difficult to translate using google translate.*
9. Pawlowski J. 2013 Reliability of the Brief Neuropsychological Assessment Instrument Neupsilin: *only Portuguese pdf version available, hence difficult to translate*
10. Ramos-Galarza C. 2019 EFECO Scale for Assessing Executive Functions in Self-Report Format: *for this study done in Ecuador, only Spanish version in pdf was available, hence difficult to translate*
11. Butman J. 2000 Spanish verbal fluency. Normative data in Argentina: *could not obtain full article online.*
12. Barreyro J. 2015 Validity and Reliability of the Running Memory Span: *only Spanish pdf version available online.*
13. Roselli Cock M. 2005 Neuropsychological Assessment of Children: a test battery for children between 5 and 16 years of age. A Colombian normative study: *only Spanish pdf version available online.*
14. Qian Y. 2011 Reliability and validity of the Chinese version of Weiss Functional Impairment Scale-Parent form for school age children: *no pdf version available at all online*
15. Dadsetan P. 2010 Kindergarten Inventory of Social/Emotional Tendencies: A cross-validation study: *no pdf available at all online*
16. Shi-jie Z. 2005 Development of the working memory battery and its validity in primary school students: *no pdf available online*
17. Lu T. 2017 Validity and reliability of the Behavior Rating Scale of Executive Function-Preschool Version parent form in China: *no pdf available online*
18. Ebrahimi A. 2016 Psychometric properties of the Behavior Rating Inventory for Executive Function-Preschool (BRIEF-P) among preschool children: *only Persian version of pdf available*
19. Andrea K. 2010 The executive functions from a neuropsychometric perspective: study from Hungary, but *no pdf available online*
20. de Oliveira A. 2014 Construction of a Scale to Assess Cognitive Planning: *study conducted in Brazil but only Portuguese pdf version available*
21. y Vila Molina G. 2010 Taylor's Figure Standardization on Mexican population: *study conducted in Mexico but only Spanish pdf version available*
22. Musso M. 2009 Assessment of executive functions in children: Analysis and adaptation of tasks in a school context: *study conducted in Argentina but only Spanish pdf version available*
23. Qian Y. 2009 Reliability and validity of the Behavior Rating Inventory of Executive Function Teacher Form for school age children in China: *no pdf available*
24. Butman J. 2000 Spanish verbal fluency test. Normative data in Argentina: *only Spanish pdf version available*
25. Abedi A. 2012 Standardization of the neuropsychological test of NEPSY on 3-4 years old children: *only Persian version of pdf available*
26. Ramos-Galarza C. 2019 EFECO scale for assessing executive functions in self-report format: *only Spanish pdf version available*
27. Musso M. 2009 Assessment of executive functions in children: analysis and adaptation of tasks in a school context: *only Spanish pdf version available*

APPENDIX III: Data Summary of Individual Instruments’ Results

**Table 3 Data Summary Chart for Number of Studies Reporting Validation of Executive Function Instruments**

| **Instrument / version** | **development study** | **Adaptation study / Content validity** | **Structural validity** | **Internal consistency** | **Cross cultural validity** | **Reliability** | **Measurement Error** | **Construct validity** | **Responsiveness** | **Total No. Studies** | **Percentage** |
| --- | --- | --- | --- | --- | --- | --- | --- | --- | --- | --- | --- |
| BRIEF: parent |  | 1 | 1 | 2 | 1 | 1 | 1 | 2 |  | 9 | 6.92 |
| BRIEF: teachers |  |  | 2 | 2 |  |  |  | 1 |  | 5 | 3.85 |
| BRIEF: personal |  |  | 1 | 1 |  |  |  |  |  | 2 | 1.54 |
| BRIEF: pre-school |  | 1 | 2 | 2 | 1 | 2 |  | 2 |  | 10 | 7.69 |
| BRIEF: TOTAL |  | 2 | 6 | 7 | 2 | 3 | 1 | 5 |  | 26 | 20 |
| ROCF |  | 1 | 1 |  | 1 | 1 |  | 1 | 1 | 6 | 4.62 |
| EFS/EFE |  | 1 | 1 | 1 |  | 1 |  |  |  | 4 | 3.08 |
| CCTT of DKEFS |  |  | 2 |  |  | 1 |  |  |  | 3 | 2.31 |
| Design fluency of DKEFS |  |  | 1 |  |  |  |  |  |  | 1 | 0.77 |
| Verbal Fluency of DKEFS |  |  | 3 |  |  | 1 |  | 1 |  | 5 | 3.85 |
| Tower Test of DKEFS |  |  | 1 | 1 |  | 1 |  |  |  | 3 | 2.31 |
| DKEFS: TOTAL |  |  | 6 |  |  | 2 |  | 1 |  | 9 | 6.92 |
| KITAP |  |  |  |  | 1 |  |  | 1 |  | 2 | 1.54 |
| JBT |  |  | 1 |  |  |  |  | 1 |  | 2 | 1.54 |
| RACER |  |  |  |  | 1 |  |  |  |  | 1 | 0.77 |
| WCST |  | 1 | 1 | 1 | 1 | 1 |  | 3 |  | 8 | 6.15 |
| Design Copying of NEPSY |  |  | 1 |  |  |  |  | 1 |  | 2 | 1.54 |
| Tower Test of NEPSY |  |  | 1 |  |  |  |  | 1 |  | 2 | 1.54 |
| Digit Span of NEPSY |  |  | 1 |  |  |  |  | 1 |  | 2 | 1.54 |
| NEPSY: TOTAL |  |  | 3 |  |  |  |  | 3 |  | 6 | 4.62 |
| Go/No-go |  | 1 | 1 | 1 | 1 | 1 |  | 1 | 1 | 7 | 5.39 |
| Letter numbering sequence |  |  | 1 |  |  | 1 |  |  |  | 2 | 1.54 |
| Shift |  | 1 |  |  | 1 | 1 |  |  | 1 | 4 | 3.08 |
| EF Touch |  |  |  |  |  |  |  | 1 |  | 1 | 0.77 |
| BASC |  |  | 1 | 1 | 1 |  |  | 1 |  | 4 | 3.08 |
| BSFT |  |  |  |  |  |  |  | 1 |  | 1 | 0.77 |
| Stroop Colour Word Test |  |  |  |  |  | 1 |  | 1 |  | 2 | 1.54 |
| SOPT |  |  |  |  |  |  |  | 2 |  | 2 | 1.54 |
| BDEFS- CA |  |  | 1 | 1 |  | 1 |  | 1 |  | 4 | 3.08 |
| TEXI |  | 1 | 1 | 1 |  | 1 |  | 1 |  | 5 | 3.85 |
| CHEXI |  | 1 | 1 | 1 |  |  |  | 1 |  | 4 | 3.08 |
| ENFEN |  |  | 1 |  |  |  |  | 1 |  | 2 | 1.54 |
| DEX |  |  | 1 |  |  |  |  |  |  | 1 | 0.77 |
| CVS of TAC |  |  |  |  |  |  |  | 1 |  | 1 | 0.77 |
| TAC |  |  | 1 |  |  |  |  |  |  | 1 | 0.77 |
| AWMA |  | 1 |  | 1 |  |  |  | 1 |  | 3 | 2.31 |
| Child Hayling Test | 1 |  |  |  |  |  |  |  |  | 1 | 0.77 |
| CANTAB |  |  |  |  | 1 |  |  | 1 |  | 2 | 1.54 |
| SFIRS | 1 |  | 1 | 1 |  | 1 |  | 1 |  | 5 | 3.85 |
| EFICA- Parents | 1 |  | 1 | 1 |  |  |  | 1 |  | 4 | 3.08 |
| EFICA- Teachers |  |  | 1 | 1 |  |  |  |  |  | 2 | 1.54 |
| EFICA: TOTAL |  |  | 2 | 2 |  |  |  | 1 |  | 5 | 3.85 |
| BES |  |  | 1 | 1 |  |  |  |  |  | 2 | 1.54 |
| PSRA |  |  |  |  | 1 | 1 |  |  |  | 2 | 1.54 |
| Computer version- Spatial span, Figure Matching Test of Hanoi and Stop Signal Task |  |  |  |  | 1 |  |  |  |  | 1 | 0.77 |
| **TOTAL** | **3** | **10** | **33** | **20** | **12** | **17** | **1** | **31** | **3** | **130** |  |

**Table 4 Data Summary Chart for Number of Studies Reporting Validation of Adaptive Function Instruments**

| **Instrument / version** | **development study** | **Adaptation study / Content validity** | **Structural validity** | **Internal consistency** | **Cross cultural validity** | **Reliability** | **Measurement Error** | **Construct validity** | **Responsiveness** | **Total No. Studies** | **Percentage** |
| --- | --- | --- | --- | --- | --- | --- | --- | --- | --- | --- | --- |
| PACS |  | 1 |  |  |  |  |  |  |  | 1 | 3.03 |
| CPAS | 1 |  | 1 | 1 |  | 1 |  | 1 |  | 5 | 15.15 |
| VABS I & II |  | 2 | 1 | 2 | 2 | 2 |  | 2 |  | 11 | 33.33 |
| CPQ |  |  | 1 | 1 |  | 1 |  | 1 |  | 4 | 12.12 |
| PADL | 1 |  | 1 | 1 |  |  |  | 1 |  | 4 | 12.12 |
| Child Function Impairment Rating Scale | 1 |  | 1 | 1 |  | 1 |  | 1 |  | 5 | 15.15 |
| IBAS |  |  |  | 1 |  | 1 |  | 1 |  | 3 | 9.09 |
| **TOTAL** | **3** | **3** | **5** | **7** | **2** | **6** |  | **7** |  | **33** |  |
